# Transfer of Neuralgia

**Published:** 1885-02

**Authors:** 


### EDITORIAL.

### Transfer of Neuralgia.

Mrs. M., in September, 1884, presented for treatment the superior first bicuspid of the left side. Upon examination, it was found to be largely decayed upon the posterior proximate surfaces, the decay occupying almost the entire side, but not extending to the pulp chamber. The tooth had not ached, and the dentine was but slightly sensitive. The pulp appeared to be sufficiently protected to warrant filling. In order to avoid any possible difficulty the cavity, after being moisted with carbolic acid dilute, was filled with oxy-phosphate of zinc. No indication of trouble occurred till about the first of December following, and then not in the tooth nor its vicinity, but in the posterior part of the head and neck, and along the upper part of the spinal column. This came on mild at first, but increased rapidly in severity, so that within a week the paroxysms were almost intolerable. They were of a true neuralgic character, striking in a moment with the greatest intensity, lasting from one to five minutes, and then passing away as promptly as they came. The pain would occur sometimes four or five times in an hour; at others several hours would pass without any pain. There was usually entire freedom from pain during the night, or at least while the patient was in bed. The pain was usually so severe that she would cry out, and the tears flow freely. For three weeks this continued, and during nearly all of this time a physician was treating the case, but without any appreciable results—at least no improvement was apparent.

After the patience of the physician had become exhausted, he suggested that, possibly, the teeth might be involved, though there were no indications that anything was wrong in that direction. This was suggested because every other cause of the difficulty seemed to be eliminated. In the latter part of December, the teeth were examined thoroughly, but without any cause being found. No tooth gave the least indication of any disease.

After fully considering the case, it was decided to remove the oxy-phosphate filling from the bicuspid above referred to. This was done without difficulty, except that when the portion of the filling covering the bottom of the cavity was raised, an intense paroxysm, in the hitherto neuralgic region, occurred, lasting for about three minutes, and then ceased as suddenly as it came. Upon the removal of the filling, an opening into the pulp chamber was plainly visible; this had been made since the filling was introduced, and through this the pulp had been irritated.

Contact through this opening exhibited the ordinary sensations of an exposed pulp, without exciting the slightest neuralgic pain. A simple application of oil of cinnamon was made to the exposed pulp, and no further pain occurred either in the tooth or elsewhere. From that time to the present, about two months, there has not been a recurrence of the neuralgia.

The pulp in the offending tooth in a few days died, but without pain, and the tooth has since been filled.

It is worthy of special note that the pulp chamber was opened *after* the tooth was filled; it certainly was not open before.

And again, that the tooth at no time after it was filled gave the slightest indication that it was the irritant which occasioned the neuralgia, and that no pain occurred in its vicinity. And again, that the neuralgic pain ceased so promptly and so absolutely after the filling was removed from the tooth.

It teaches an important lesson for both physician and dentist. The physician should not permit a patient to suffer on for weeks, in intense pain, the cause of which he does not know, and which he is powerless to relieve, without suggesting an examination of the teeth, and indeed, the mouth as a whole.

It teaches the dentist that difficulties, and serious ones, too, may arise from very unexpected sources; and that too much care and discrimination cannot be exercised in cases where there is any doubt or obscurity; and that oftentimes the removal of an irritant is the proper course rather than persistent medication.

There are, doubtless, many cases of neuralgia dependent upon diseased teeth that seem very obscure to the physician, that may be readily detected and removed by the intelligent dentist. Nervous affections of all sorts should be thoroughly studied by every one who attempts to treat the teeth.

